# Does the pleth variability index have any predictive value for intraoperative respiratory problems in bariatric surgery?

**DOI:** 10.55730/1300-0144.5771

**Published:** 2023-12-14

**Authors:** Esra ÖZAYAR, Mehmet ŞAHAP, Handan GÜLEÇ, Aysun KURTAY, Adem SELVİ, Hakan BULUŞ, Özlem ARI

**Affiliations:** 1 Department of Anesthesiology and Reanimation, Faculty of Medicine, University of Health Sciences, Ankara, Turkiye; 2 Department of Anesthesiology and Reanimation, Faculty of Medicine, Yıldırım Beyazıt University, Ankara, Turkiye; 3 Department of Anesthesiology and Reanimation, Ankara Ataturk Sanatorium Training and Research Hospital, Ankara, Turkiye; 4 Department of General Surgery, Faculty of Medicine, University of Health Sciences, Ankara, Turkiye

**Keywords:** Bariatric, pleth variability index, respiratory

## Abstract

**Background/aim:**

We aimed to search the relationship between the preoperative PVI (pleth variability index) and intraoperative respiratory parameters to reveal whether PVI can be used as a prediction tool in bariatric surgery.

**Materials and methods:**

Forty patients undergoing bariatric surgery were included. Noninvasive pleth variability index measured via finger probe before induction of general anesthesia. Following intubation each patient was ventilated in controlled mode. Intraoperative blood pressure, peak airway pressure, end-tidal CO_2_, SpO_2_, PEEP, and FiO_2_ were recorded every 5 min for the first 10 min and then every 10 min until extubation. Steroid and bronchodilator requirements were recorded.

**Results:**

The systolic pressure-PVI, oxygen saturation-PVI relationship was statistically significant (p = 0.03, p = 0.013). A relationship was found between pleth variability index and peak airway pressure (p = 0.002). No correlation was detected between end-tidal CO_2_ and pleth variability index. The relationship between steroid, bronchodilator use, and PVI was significant (p = 0.05, p = 0.01). A positive correlation between PEEP and PVI was detected at varying time points. A positive correlation was found between FiO_2_-PVI.

**Conclusion:**

A relationship was found between PVI and intraoperative peak airway pressures, oxygen saturation, PEEP, bronchodilatator, and steroid usage. This result may be inspiring to conduct larger studies addressing the issue of predicting intraoperative respiratory problems in bariatric surgeries.

## 1. Introduction

Obesity stands out as a significant risk factor for the respiratory system, contributing to various physiological changes. The presence of substantial adipose tissue in the thoracic and abdominal regions not only alters the mechanical properties of the lungs but also introduces inflammatory cytokines from adipose tissue, correlating with impaired lung function [1,2]. In the preoperative context of bariatric surgery, anesthesiologists grapple with concerns about potential respiratory issues due to these altered lung physiology factors [2].

Research indicates that the hyperinflammation observed in obstructive airway diseases and reactive airway diseases manifests in a heightened degree of pulsus paradoxus (PP) [3]. The Pleth Variability Index (PVI) (Masimo Corp., Irvine, CA), serving as a surrogate measure of pulsus paradoxus, employs an algorithm for automated and continuous calculation of respiratory variations in the pulse oximeter waveform amplitude [4,5]. While existing studies primarily focus on PVI in the context of fluid responsiveness, there is a notable gap in trials exploring the relationship between preoperative PVI and respiratory parameters [5]. Motivated by this gap, we conducted a prospective study to scrutinize the link between preoperative PVI and intraoperative respiratory parameters and challenges. We aim to discern the potential utility of preoperative PVI as a predictive tool for intraoperative respiratory outcomes in bariatric surgeries.

## 2. Materials and methods

The study protocol received ethical approval from the Institutional Review Board for human subjects (15/1722). Following the acquisition of written informed consent, we enrolled 40 consecutive patients undergoing sleeve gastrectomy. Inclusion criteria comprised age between 18–60, body mass index (BMI) >30 kg/m2, and American Society of Anesthesiologists physical status I-II. Patients with lung diseases (chronic obstructive pulmonary disease (COPD), asthma), cardiac diseases (congestive heart failure, constrictive pericarditis, pulmonary hypertension), chronic renal diseases, and endocrine diseases that could affect intravascular fluid status were excluded.

During the perioperative 12-h fasting period, patients received 1000 mL saline infusion intravenously to standardize intravascular volume status. No premedication was administered in the preoperative area. Upon arrival at the operating room, patients were positioned on an operating table, and standard monitoring, including electrocardiogram (ECG), noninvasive blood pressure (NIBP), and oxygen saturation (SpO_2_), was performed. Before the induction of general anesthesia, a Masimo Radical-7. A set pulse oximeter (Masimo Corp., Irvine, CA) was applied to the right-hand index finger, contralateral to the blood pressure cuff. To prevent interference with signal monitoring, precautions were taken due to strong ambient lighting. A third-year resident, blinded to the study to prevent any prejudgment in clinical decision-making, measured and recorded the PVI. Patient charts were meticulously reviewed for age, BMI, sex, FEV1, and FEV1/FVC information collected during preoperative preparation.

Induction was achieved with lidocaine (1 mg/kg, ideal body weight (IBW)), propofol (2.5 mg/kg, IBW), fentanyl (1 μg/kg, lean body weight (LBW)), and rocuronium bromide (0.6 mg/kg, IBW). After tracheal intubation, each patient was ventilated in a controlled mode. Following our institution’s protocol, mechanical ventilation parameters were set as follows: tidal volume 7 mL/kg (IBW), I:E ratio of 1:2, PEEP: 6 cm H_2_O, inspiratory O_2_ fraction (FiO_2_) 0.5, respiratory rate of 12 breaths/min targeting an end-tidal CO_2_ value of approximately 35–40 mmHg. Intraoperative parameters, including blood pressures, peak airway pressures, end-tidal CO_2_, SpO_2_, PEEP, and FiO_2_, were recorded every 5 min for the first 10 min and then every 10 min until extubation. The anesthesiologist, blinded to the study, was permitted to adjust mechanical ventilator settings to maintain oxygen saturation over 90%. Steroid and bronchodilator medications used for respiratory problems were documented.

Descriptive statistics for study variables were expressed as the mean (standard deviation) or frequency (percentage). Spearman correlation coefficient was used to calculate correlations of variables with PVI. Logistic regression was employed to investigate the relationship between PVI and steroid and bronchodilators, with odds ratios and 95% confidence intervals provided. According to post hoc power analysis of saturation and peak airway pressure, the power was 0.88, 0.91, and 0.93, 0.95 at the last and first two time points, respectively. Analyses were conducted using the SAS University Edition 9.4 program, and a significance level of p < 0.05 was considered.

## 3. Results

**Figure 1:**
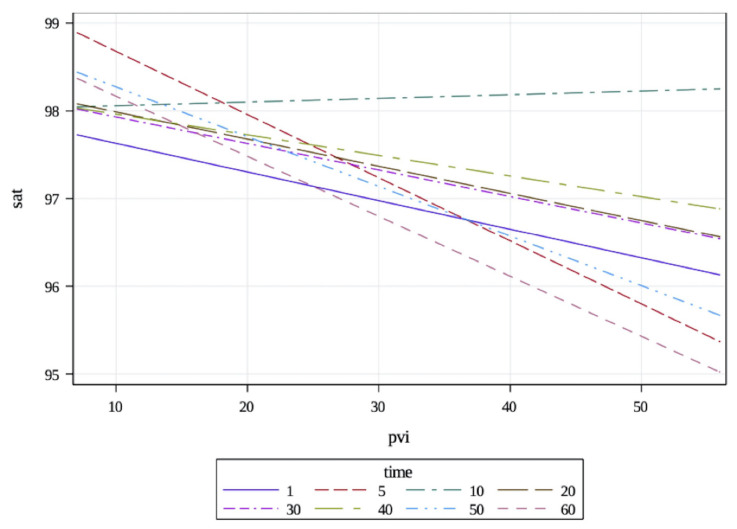
The relationship between oxygen saturation (SpO_2_) and pleth variability index (PVI). The relations between SpO_2_ and PVI were always negative, except for the 10th min.

A total of 17 male and 23 female patients participated in the study (Tables 1–2). When examining the relationship between PVI and SpO_2_, a statistically significant result was observed (p = 0.013), with varying relationships at different time points. The graphical representation of this relationship is depicted in Figure 1. The saturation consistently decreased by 0.07, 0.03, 0.03, 0.02, 0.05, and 0.07 units when PVI increased by 1 unit at time points 5, 20, 30, 40, 50, and 60, respectively. Except for the 10th min, the relationship between PVI and saturation was consistently negative, indicating a negative correlation.

**Figure 2:**
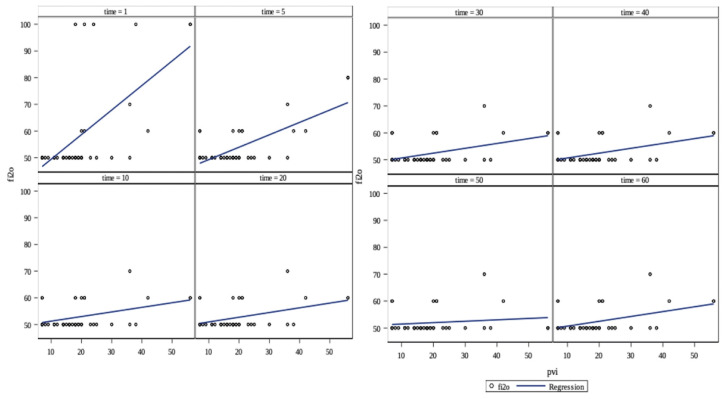
The relationship between FiO_2_ and pleth variability index (PVI). A significant relationship was found between PVI and FiO_2_, p = 0.0004, but this relationship was different at different time points, p < 0.0001.

**Figure 3:**
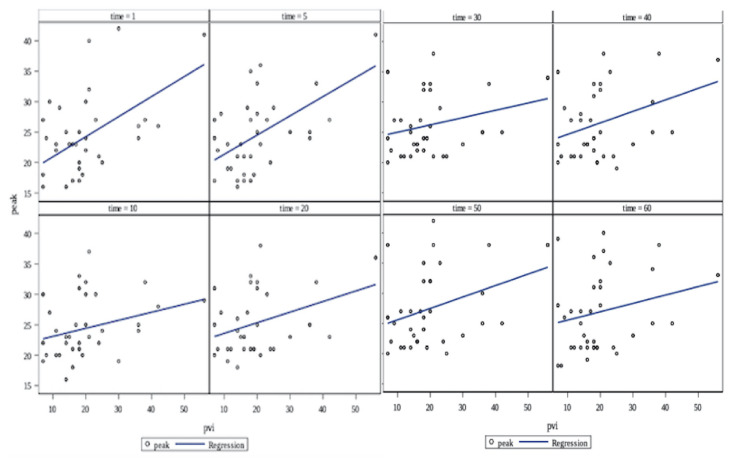
The relationship between peak airway pressure and PVI. A significant relationship was found between PVI and peak airway pressure, p = 0.002. However, this relationship was different at different times, p = 0.0014.

In the case of PVI and FiO_2_, a positive correlation was identified (p = 0.0004), as illustrated in Figure 2. FiO_2_ increased by 0.92, 0.46, 0.17, 0.18, 0.18, 0.18, 0.05, and 0.18 units when PVI increased by 1 unit at the 1st, 5th, 10th, 20th, 30th, 40th, 50th, and 60th time points, respectively. Significant associations were found between PVI and peak airway pressure (p = 0.002), as shown in Figure 3. Peak airway pressure increased by 0.33, 0.32, 0.13, 0.17, 0.12, 0.19, and 0.14 units when PVI increased by 1 unit at the 1st, 5th, 10th, 20th, 30th, 40th, 50th, and 60th time points, respectively.

**Figure 4:**
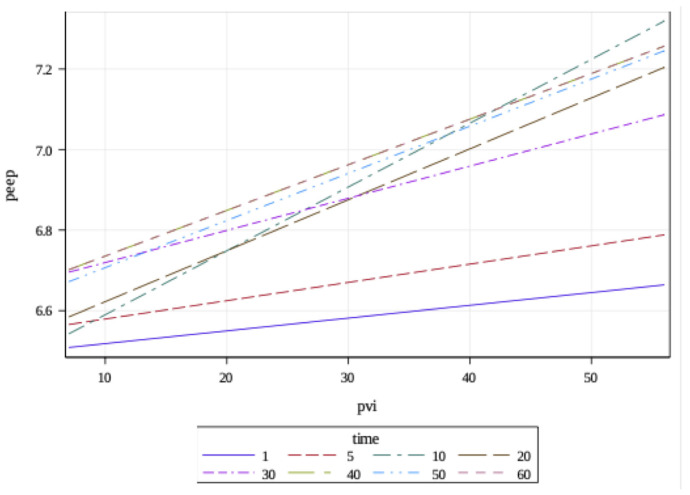
The relationship between positive end expiratory pressure (PEEP) and pleth variability index (PVI). A positive correlation found between PEEP and PVI.

A time-varying positive correlation was observed between PVI and positive end-expiratory pressure (PEEP), as depicted in Figure 4. PEEP increased by 0.003, 0.0005, 0.016, 0.013, 0.008, 0.01, 0.01, and 0.01 units when PVI increased by 1 unit at the 1st, 5th, 10th, 20th, 30th, 40th, 50th, and 60th time points, respectively.

In investigating the relationship between PVI and steroid usage, an odds ratio (OR) of 1.065 (0.99–1.135) was found with a p-value of 0.05. In other words, when PVI increased by 1 unit, the odds of being steroid-positive increased by 6%. Similarly, a relationship was established between PVI and bronchodilator usage, with a p-value of 0.01. No correlation was observed between end-tidal CO_2_ and PVI. The correlation coefficient between PVI and forced expiratory volume in one second (FEV1) was −0.286 (p = 0.07). Additionally, the systolic pressure-PVI relationship was statistically significant (p = 0.03)

## 4. Discussion

The existing literature underscores the elevated risk of intraoperative respiratory complications in obese patients [6,7]. The prediction of such complications in the context of bariatric surgery remains an essential area warranting further investigation. Obesity induces respiratory functional disturbances, including gas exchange alterations, reduced respiratory system compliance, heightened work of breathing, and increased small airway resistance [8]. Accumulation of thoracic and abdominal fat compresses the lungs, reducing lung volumes, while the traction of lung parenchymal attachments around the airways may lead to airway collapse. In addition to mechanical alterations, metabolic factors associated with obesity contribute to adverse effects on lung function. Notably, obesity is a key factor in airway hyperinflammation and hyperresponsiveness, potentially resulting in challenging intraoperative airway problems [9]. While perioperative complications are more prevalent in obese patients than in nonobese individuals, there are also specific complications linked to the pathophysiological state of obese patients [10–12].

In our study, our objective was to identify a predictive tool for recognizing intraoperative airway problems, which are not uncommon in bariatric surgery. We initiated our investigation based on the relationship between obesity, the increased prevalence of pulsus paradoxus (PP), and the connection between PP and respiratory dynamics [5,13–15]. Unlike previous studies that focused on PVI and obstructive airway diseases, our study is the first to explore the relationship between PVI and intraoperative airway problems in obesity surgery [3,5].

Brandwein et al. reported in their study that PVI might serve as a useful triage tool for children with obstructive airway disease in the emergency department, finding that higher PVI values correlated with the severity of the disease [3]. In our study, we established a relationship between PVI and SpO2, as well as peak airway pressure. Higher PVI values were associated with elevated peak airway pressures and decreased saturation. Additionally, patients with higher PVI values required more frequent administration of steroids and bronchodilators.

Tassoudis et al. investigated the incidence of bronchospasm in obese patients compared to nonobese patients, concluding that the incidence of bronchospasm was higher in obese individuals undergoing elective laparoscopic surgery. In their trial, peak airway pressures in obese patients were also higher than in nonobese patients [7]. Although we did not directly compare peak airway pressures with nonobese patients, our study, focused on bariatric surgery population, revealed that peak airway pressure increased with higher PVI values.

Desebbe et al. [16] explored the ability of PVI to predict the hemodynamic effects of positive end-expiratory pressure (PEEP) in mechanically ventilated patients, demonstrating that PVI is influenced by tidal volumes and PEEP. Notably, their study involved patients who were sedated and mechanically ventilated in volume-controlled mode, whereas our study involved PVI measurements before the induction of anesthesia, without sedatives or ventilation support that could influence PVI values.

Despite these contributions, our study has some limitations. Factors such as pneumoperitoneum and the degree of Trendelenburg position can influence ventilation parameters, oxygenation, and peak airway pressures. Although we did not record these factors, the surgical team adhered to a consistent procedure for intraabdominal pressure and Trendelenburg degree for all patients. Additionally, being a medium-sized medical center, the number of patients in our study is not high.

## 5. Conclusion

While numerous studies have explored the use of PVI to evaluate fluid responsiveness, our study suggests an additional role for PVI as a promising tool to predict perioperative respiratory problems in the bariatric surgery population. Larger studies with a higher number of patients would be valuable to establish a more robust correlation between PVI and intraoperative respiratory complications.

## Figures and Tables

**Table 1:** Demographic variables.

		n	%
**Sex**	**Male**	17	42.5
	**Female**	23	57.5
**Comorbidities**	**Present**	26	65.0
	**None**	14	35.0

**Table 2:** Findings according to demographic variables.

	Minimum	Maximum	Mean	Standard deviation
**Age**	20.00	55.00	35.33	10.05
**BMI**	40.00	62.00	45.43	4.88
